# Tyrosine kinase inhibitors show different anti-brain metastases efficacy in NSCLC: A direct comparative analysis of icotinib, gefitinib, and erlotinib in a nude mouse model

**DOI:** 10.18632/oncotarget.21936

**Published:** 2017-10-19

**Authors:** Jianlong Tan, Min Li, Wen Zhong, Chengping Hu, Qihua Gu, Yali Xie

**Affiliations:** ^1^ Department of Respiratory Medicine, Xiangya Hospital, Central South University, Changsha, Hunan 410008, China

**Keywords:** non-small cell lung cancer (NSCLC), tyrosine kinase inhibitors, brain metastasis, blood-tumor barrier, cerebrospinal fluid

## Abstract

Brain metastasis is an increasing problem in non-small cell lung cancer (NSCLC) patients. Tyrosine kinase inhibitors (TKIs), including gefitinib, erlotinib, and icotinib, are reported to be effective in patients with brain metastases. However, direct comparative studies of the pharmacokinetics and efficacy of these three drugs in treating brain metastases are lacking. In the present investigation, we found that gefitinib penetrated the blood-tumor barrier and was distributed to brain metastases more effectively than erlotinib or icotinib in a nude mouse model. The 1-h ratio of brain metastases to plasma concentration for gefitinib, erlotinib, and icotinib was 9.82±1.03%, 4.83±0.25%, and 2.62±0.21%, respectively. The 2-h ratio of brain metastases to plasma concentration for gefitinib, erlotinib, and icotinib was 15.11±2.00%, 5.73±1.31%, and 2.69±0.31%, respectively. Gefitinib exhibited the strongest antitumor activity (*p*_gefitinib vs. erlotinib_=0.005; *p*_gefitinib vs. icotinib_=0.002). Notably, erlotinib exhibited a better treatment efficacy than icotinib (*p*=0.037). Consistently, immunohistochemical data showed that TKIs differentially inhibit the proliferation of metastatical tumor cells. Gefitinib and erlotinib markedly inhibited the proliferation of tumor cells, while there were more ki-67-positive tumor cells in the icotinib group. Additionally, gefitinib inhibited the phosphorylation of EGFR better than the other drugs, whereas pEGFR expression levels in erlotinib groups were lower than levels in the icotinib group (*p*_gefitinib vs. erlotinib_=0.995; *p*_gefitinib vs. icotinib_=0.028; *p*_erlotinib vs. icotinib_=0.042).Altogether, our findings suggest that gefitinib and erlotinib can inhibit the growth of PC-9-luc brain tumors. Gefitinib demonstrated better antitumor activity and penetration rate in brain metastases than erlotinib or icotinib.

## INTRODUCTION

Mutations in epidermal growth factor receptor (EGFR) gene activation (19 exon del/L858R) may be a risk factor for brain metastases in patients with non-small cell lung cancer (NSCLC) [1]. Approximately 26% of patients who receive clinical benefit from EGFR tyrosine kinase inhibitor (TKI) treatment develop central nervous system (CNS) metastases, including in the brain parenchyma and pia mater, once clinical control of the primary pulmonary tumor foci is achieved [2]. The invasiveness of EGFR mutations [3], prolonged survival [2], and blood-brain barrier (BBB) blockage of drug penetration [4] are potential factors that may increase the occurrence of brain metastases in EGFR mutation-positive NSCLC. Once brain metastases occur, patient prognosis remains poor, even if the patient receives clinical benefit from the use of active and comprehensive treatment, such as whole-brain radiotherapy (WBRT), stereotactic radiotherapy, surgical resection, or chemotherapy [5].

In metastatic brain tumors, the BBB and blood-tumor barrier (BTB) [6] limit the amount of antitumor drug that can penetrate the tumor tissue, thereby resulting in ineffective treatment at the tumor site. The therapeutic effects of traditional chemotherapy drugs on brain metastases are not sufficient [7], and more effective drugs are urgently required. Compared with traditional chemotherapy drugs, EGFR TKIs have a lower molecular weight and good cellular permeability. Research has shown that they are more effective in treating NSCLC than traditional chemotherapy drugs [8]. Gefitinib or erlotinib monotherapy at the normal dose can improve symptoms in NSCLC patients with brain metastases and prolong survival [9, 10]. The EGFR TKI icotinib, alone or in combination with WBRT, can significantly extend median survival times in NSCLC patients with brain metastases, with a response rate of up to 80% [11, 12]. When intracranial metastatic lesions reprogress after treatment with a normal dose of EGFR TKIs, some researchers advocate using continuous, intermittent, or high-dose methods of administration. Some patients show improvement using these methods, indicating that a high dose of EGFR TKIs can increase the concentration of the medication in cerebrospinal fluid (CSF). This shows that the main issue limiting drug efficacy is the distribution of EGFR TKIs in the brain, rather than resistance [13–15]. Furthermore, some patients develop CNS lesions when using gefitinib and benefit from switching to erlotinib [16, 17]. Small-sample comparison studies have shown that the concentration of erlotinib in human CSF is significantly higher than that of gefitinib, which suggests that gefitinib and erlotinib possess unequal ability to penetrate the BBB [4]. There may also be differences in the efficacy of various EGFR TKIs in treating brain metastases.

Clinically, drug concentration in the CSF is commonly used as an important criterion in evaluating drug efficacy [4]. However, owing to the existence of the BTB, there is no linear correlation between drug concentrations in CSF and brain parenchyma or intracranial tumor lesions [18]. Although the brain parenchyma also displays a significant difference in drug concentrations in lesions and in normal brain tissue at the tumor periphery, the difference may be related to the integrity of the BTB and efflux pumps, such as P-glycoprotein (P-gp/ABCB1) and breast cancer resistance protein (BCRP/ABCG2) [19]. Therefore, in this study, an NSCLC brain metastasis mouse model was constructed, and clinically administered dosages were simulated to comparatively study the efficacy of gefitinib, icotinib, and erlotinib, along with plasma, CSF, brain tissue, and brain tumor tissue concentrations in NSCLC brain metastases.

## RESULTS

### Gefitinib and erlotinib show antitumor activity on lung cancer brain metastases in nude mice

**Figure 1:**
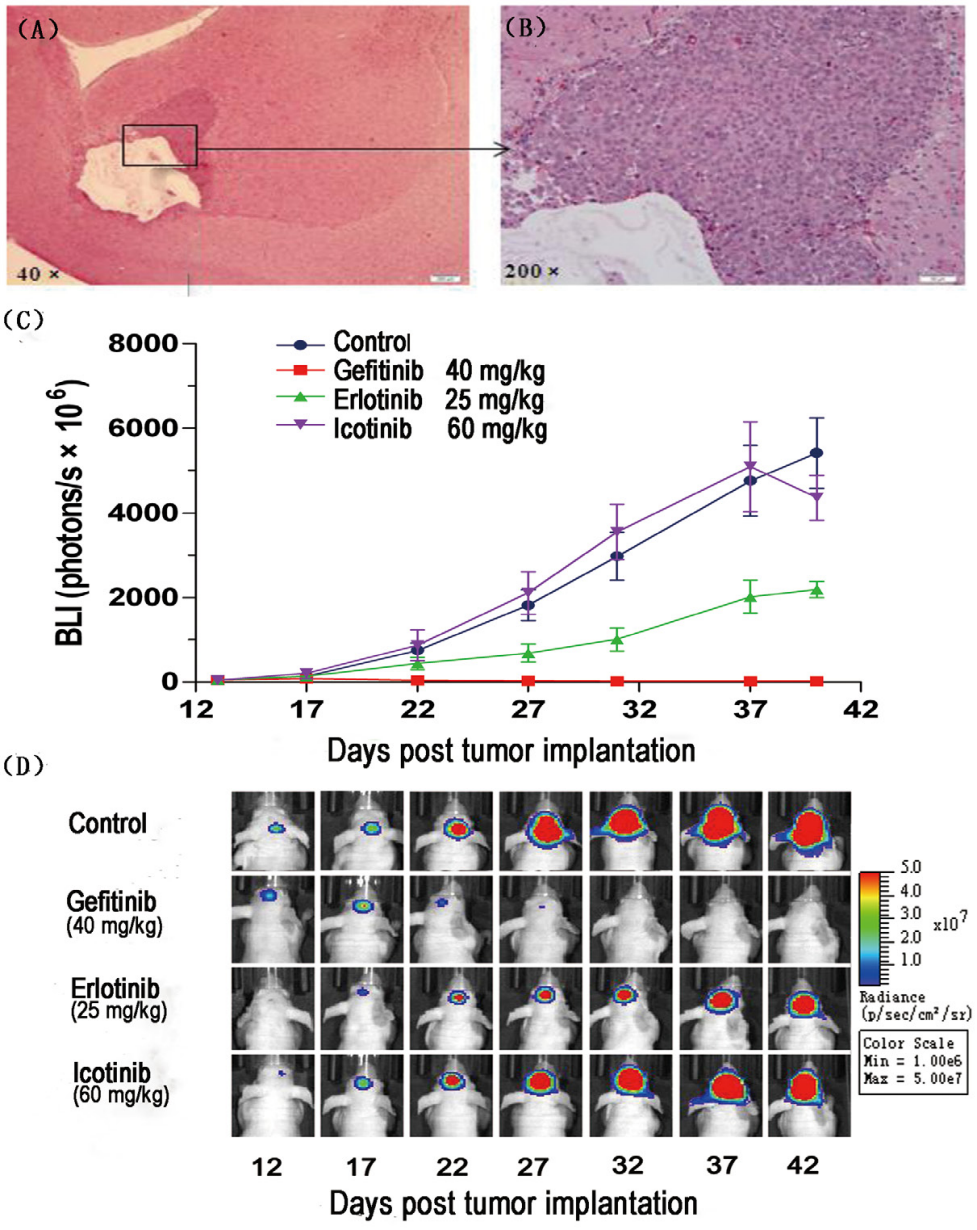
Brain metastasis model of PC-9-luc cells and efficacy of EGFR tyrosine kinase inhibitors PC-9-luc cell were inoculated into the cerebrum of BALB/c nude mice. **(A)** Macroscopic appearance of brain tumors. **(B)** Microscopic appearance of brain lesions (×200). **(C)** Mice were treated orally with control (n=3), 40 mg/kg gefitinib (n=3), 25 mg/kg erlotinib (n=3), or 60 mg/kg icotinib (n=3). Treatment was administered daily on days 13–40. Luminescence was evaluated twice per 10 days and before sacrifice. Bars indicate standard error. **(D)** Images of representative mice are shown in the top row.

In our first set of experiments, we set out to determine the efficacy of the three EGFR-TKIs in mice bearing intracranially implanted tumors. Histopathological examinations confirmed the presence of tumors in the cerebra (Figure 1A, 1B) Dynamic changes in the signal intensity of intracranial tumors were evaluated by bioluminescence imaging to assess the efficacy of individual therapeutics (Figure 1C). As expected, the highest change from baseline in bioluminescence was observed in negative control-treated animals (from 46 to 5,412 × 10^6^ photons/s on average). Compared with that in the control group, metastatic tumor burden did not increase after the start of gefitinib treatment, and the average bioluminescence value of tumors in the gefitinib treatment group was 24 × 10^6^ photons/s on the final day of the study (*p*=0.002). The average bioluminescence value in the erlotinib treatment group was 2,187 × 10^6^ photons/s (*p*=0.029). The average bioluminescence value of tumors in the icotinib treatment group was 4,356 × 10^6^ photons/s (*p* = 0.851). Interestingly, the erlotinib group showed better drug efficacy than the icotinib group (*p*=0.037). Moreover, compared with erlotinib and icotinib, gefitinib showed higher antitumor activity (*p*_gefitinib vs. erlotinib_=0.005; *p*_gefitinib vs. icotinib_=0.002; Figure 1D). These data suggested that gefitinib was highly effective against brain lesions.

### Gefitinib results in a higher ratio of brain tumor to plasma concentration than erlotinib and icotinib

To further investigate the role of the BBB and BTB in limiting EGFR-TKI distribution, EGFR-TKI concentrations were determined in normal brain, brain tumors, CSF, and plasma. Samples were collected 1, 2 and 24 h after EGFR-TKI administration on day 40 after tumor implantation. Based on our preliminary experiments, sampling time points were selected to correspond to blood C_max_ 1 and 2 h with a once-daily oral dosing schedule. The dose was simulated to correspond to clinically administered doses; conversion was based on body surface area.

**Figure 2:**
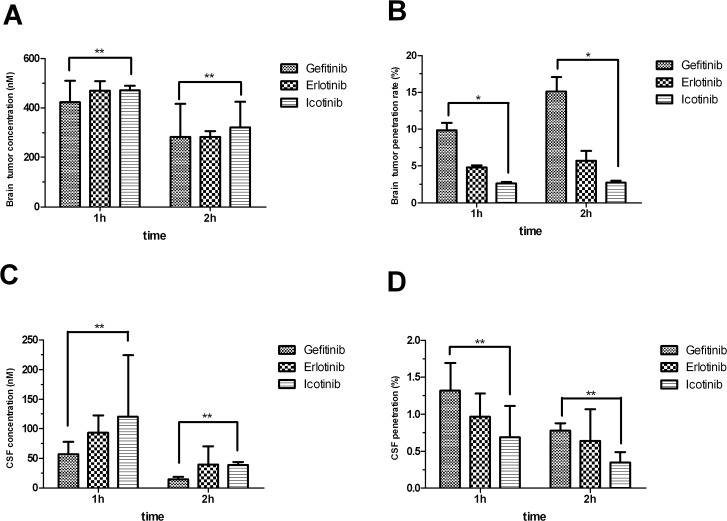
Brain tumor concentrations **(A)** and penetration rates **(B)** shown as mean ± SD. Cerebrospinal fluid (CSF) concentrations **(C)** and penetration rates **(D)** shown as mean ± SD. The brain tumor penetration of gefitinib is significantly higher than that of erlotinib or icotinib. ^*^p<0.05, ^**^p>0.05.

Drug concentrations and penetration rates in plasma, CSF, and tumor tissue after continuous gavage treatment with erlotinib (25 mg∙ kg^−1^), gefitinib (40 mg∙kg^−1^), and icotinib (60 mg∙kg^−1^) are shown in Table 1. The tumor tissue penetration rate of gefitinib after 1 and 2 h was significantly higher than that of erlotinib and icotinib (1 h: *p*_gefitinib vs. erlotinib_=0.023, *p*_gefitinib vs. icotinib_=0.011, *p*_erlotinib vs. icotinib_=0.001; 2 h: *p*_gefitinib vs. erlotinib_<0001, *p*_gefitinib vs. icotinib_<0.001, *p*_erlotinib vs. icotinib_=0.037). The 24-h brain tumor tissue concentration of gefitinib was higher than that of erlotinib (62.89 nM vs. 1.02 nM). There was no statistically significant difference between 1-h and 2-h CSF drug concentrations and penetration rates of the three drugs (Figure 2). We surmise that the different ratio of brain tumor to plasma concentration explains the difference in efficacy between gefitinib, erlotinib, and icotinib.

**Table 1:** Drug concentrations and penetration rates of erlotinib, gefitinib, and icotinib in plasma, brain tissue, brain tumor tissue, and cerebrospinal fluid (CSF) in the lung adenocarcinoma brain metastases model

Time (h)	1	2	24
Gefitinib			
C_plasma_ (nM)	4272.18±465.49	1887.48±830.44	48.00^*^
C_brain tumor_ (nM)	422.05±87.99	283.02±132.20	62.89^*^
C_normal brain_ (nM)	343.57±71.44	224.93±108.39	19.82^*^
C_CSF_ (nM)	56.98±21.21	14.21±4.66	BLOQ
C_brain tumor_/C_plasma_ (%)	9.82±1.03	15.11±2.00	131^*^
C_CSF_/C_plasma_ (%)	1.32±0.37	0.78±0.10	NA
Erlotinib			
C_plasma_ (nM)	9710.00±284.18	5024.42±769.37	BLOQ
C_brain tumor_ (nM)	469.88±37.43	258.12±45.52	1.02^*^
C_normal brain_ (nM)	223.75±43.20	134.85±17.07	BLOQ
C_CSF_ (nM)	93.44±29.33	39.50±31.26	BLOQ
C_brain tumor_/C_plasma_ (%)	4.83±0.25	5.73±1.31	NA
C_CSF_/C_plasma_ (%)	0.97±0.31	0.64±0.43	NA
OSI-420			
C_plasma_ (nM)	1346.58±137.13	688.51±67.42	BLOQ
C_brain tumor_ (nM)	20.27^*^	11.33±2.48	0.92^*^
C_normal brain_ (nM)	15.10±3.58	7.77±1.59	0.64^*^
C_CSF_ (nM)	5.28^*^	6.93^*^	BLOQ
C_brain tumor_/C_plasma_ (%)	1.54^*^	1.63±0.23	NA
C_CSF_/C_plasma_ (%)	0.39^*^	1.10^*^	NA
Icotinib			
C_plasma_ (nM)	18130.46±2160.02	11763.41±2805.72	BLOQ
C_brain tumor_ (nM)	471.32±18.74	321.26±103.39	BLOQ
C_normal brain_ (nM)	303.82±41.34	162.80±51.84	BLOQ
C_CSF_ (nM)	120.66±104.02	38.56±5.12	BLOQ
C_brain tumor_/C_plasma_ (%)	2.62±0.21	2.69±0.31	NA
C_CSF_/C_plasma_ (%)	0.69±0.42	0.35±0.14	NA

All concentration values are expressed as means ± standard deviation, n=3. BLOQ: Below the limit of quantification; ^*^: total concentration in mouse tissue was below the limit of quantification, therefore n < 3, cannot calculate SD; NA: not calculated.

### Gefitinib and erlotinib treatment differentially inhibits the proliferation of metastatical tumor cells and phosphorylation of EGFR

**Figure 3:**
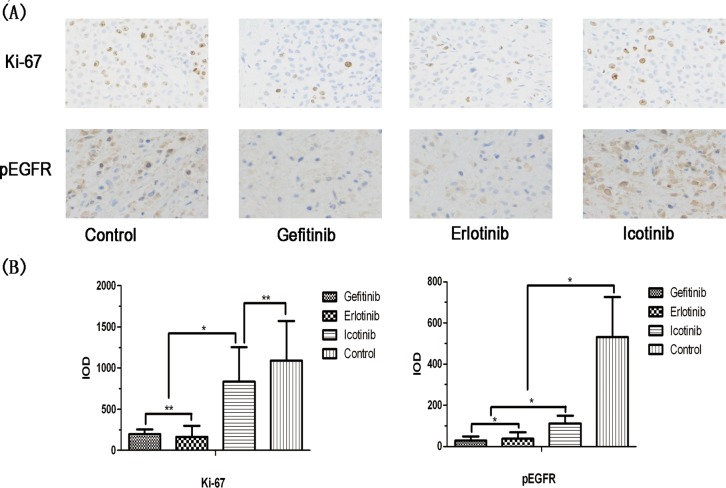
Comparison of immunohistochemistry findings for gefitinib, erlotinib, and icotinib Brain tumor specimens were collected 24 h after administration of each marker. **(A)** Intensity of Ki-67 and pEGFR in erlotinib, gefitinib, and icotinib tumor tissues (×200, light microscopy). **(B)** Significant inhibition of cell proliferation in the gefitinib and erlotinib groups. Ki-67 expression was lower than in the icotinib and control groups. pEGFR immunohistochemistry scores in the intervention groups were significantly lower than in the control group; pEGFR scores in the gefitinib and erlotinib groups were lower than in the icotinib group (*p*_gefitinib vs. erlotinib_=0.955; *p*_gefitinib vs. icotinib_=0.028; *p*_erlotinib vs. icotinib_=0.042).^*^p<0.05,^**^p>0.05.

The protein Ki-67 is a marker of cell proliferation. pEGFR is a byproduct of EGFR activity, and can serve as a preliminary indicator of the efficacy of EGFR TKIs against tumor cells. Immunohistochemistry analysis of Ki-67 and pEGFR in brain tumor tissues showed that compared with the negative control group (integrated optical density [IOD] value 1,092.62±480.72), gefitinib and erlotinib inhibited Ki-67 expression (IOD values: 198.55±56.67 and 163.37±139.51, respectively [*p*<0.05]). In the icotinib group, the Ki-67 IOD value was 833.94±417.87, which indicates higher expression than in the gefitinib or erlotinib groups (*p*<0.05). However, there was no statistically significant difference compared with the negative control group. Immunohistochemistry findings in pEGFR showed that the IOD value in the negative control group was 532.12±192.36, while the IOD values of the gefitinib, erlotinib, and icotinib groups were 27.72±20.21, 36.65±32.19, and 109.77±38.61, respectively (*p*<0.05 compared with the negative control group). pEGFR expression levels in the gefitinib and erlotinib groups were lower than levels in the icotinib group (*p*_gefitinib vs. erlotinib_=0.995; *p*_gefitinib vs. icotinib_=0.028; *p*_erlotinib vs. icotinib_=0.042; Figure 3). Those data suggested that TKIs differentially inhibit the proliferation of metastatical tumor cells and phosphorylation states of EGFR.

## DISCUSSION

Brain metastases are a common and serious complication associated with tumors. Approximately 25–30% of NSCLC patients experience CNS metastases [20]. EGFR TKIs have become a first-line treatment for EGFR mutation-positive NSCLC patients; they are also used in NSCLC patients with brain metastases, and numerous studies have confirmed their safety and efficacy [9, 10]. However, there is a lack of randomized controlled trials investigating how the three commonly used EGFR TKIs differ in regards to efficacy in treating brain metastases. Therefore, this study adopted a strict randomized controlled trial design in constructing an NSCLC brain metastasis animal model, and simulated clinical doses to compare the efficacy of the three EGFR TKIs in treating NSCLC brain metastases. A comparison study of the efficacy and concentrations of the three EGFR TKIs in plasma, tumor tissue, and CSF in an NSCLC brain metastases model has not yet been reported.

In this study, gefitinib and erlotinib both showed activity against metastatic brain tumor cells; gefitinib showed a stronger activity than erlotinib, while icotinib's activity was similar to that of the control group. Heimberger et al. found that gefitinib can significantly prolong median survival time in mice with brain metastases in a dose-dependent manner [21]. In clinical studies of treatment schemes using gefitinib or erlotinib as first-line therapy, the objective efficacy rate was between 55–83% [22]. Zhang et al. [23] compared gefitinib and erlotinib in EGFR mutation-positive NSCLC patients with brain metastases. Patients who received gefitinib exhibited longer progression-free survival than those who received erlotinib, although there were no significant differences in efficacy. This result differs from that noted by Togashi et al. [4], who reported an efficacy rate of 57.14% (four out of seven cases) in the treatment of NSCLC with brain metastasis using erlotinib; this was higher than the efficacy rate for gefitinib (33.3%, one out of three cases). However, studies with larger sample sizes are required to confirm these efficacy rates. Furthermore, Choong et al. [24] reported a striking response to gefitinib in a patient with leptomeningeal metastases and erlotinib-refractory lung adenocarcinoma. In short, the efficacy of EGFR-TKIs in the treatment of brain metastases reported in various studies differ greatly. The results of this study indicate that further studies could be made for the comparison.

There are few similar reports on icotinib, although one brain metastasis patient with EGFR mutation had a survival time of over 1 year after receiving icotinib monotherapy [25]. In NSCLC patients with brain metastases treated with icotinib, 12 EGFR mutation patients had a median survival time of 21.2 months, showing that icotinib can be beneficial for NSCLC patients with brain metastases [26]. The differences between this study and the clinical results may be related to the different pharmacokinetics and pharmacodynamics between species [27]. This may not be absolutely consistent with clinical situations. To assess the concentrations of the drug, a decrease in optical signal intensity, as well as pEGFR and Ki-67 expression, were used to reflect efficacy, rather than using overall survival as the final observation indicator. The optical signal intensity and pEGFR and Ki-67 expression were lower in the icotinib group than in the control group; the efficacy of icotinib may therefore be identified more accurately with longer treatments.

The primary reason for the difference in efficacy among the three drugs may have been their ability to penetrate the BTB. In this study, the 1-h and 2-h penetration rates of gefitinib were significantly higher than those of erlotinib and icotinib. Similar to the BBB, the BTB strongly influences the distribution of medication within lesions. Medication passes through the BTB by passive diffusion and active transport, and the integrity of the BTB has an effect on passive diffusion. Active transport is related to the efflux pump on the BTB [19]. In metastatic brain tumors, although the integrity of the BTB is destroyed, the active efflux mechanism remains intact. Studies have indicated that the outflow capacity of the BTB is similar to that of the normal BBB [28]. The types of efflux transporters on the BTB and their mechanisms are unclear. Some researchers observed expression of P-gp in the BTB in a brain metastasis model of breast cancer brain metastasis model [28, 29]. Adkins et al. [28] found that the normal expression level of P-gp in the BTB was very close to that of the BBB, which significantly influences the ability of drugs to enter the tumor. In this study, we found a difference in drug concentrations in normal brain tissue and brain tumor tissue. Similarly, Sharma et al. [30] observed differences in the concentrations of gefitinib in peritumoral tissue and tissue in the central tumor mass in a brain glioma mouse model, and confirmed that the concentration difference was related to P-gp expression. Gefitinib and erlotinib have both been identified as substrates of P-gp, and are both subject to P-gp and BCRP efflux, which influences their distribution in the brain [31, 32]. This difference in penetration rates may be associated with the strength of the effect the efflux pump has on different drugs. Therefore, the effects of P-gp and other efflux pumps on the three EGFR TKIs we tested should be investigated further under the same experimental conditions.

Differences in the tissue clearance rates of the three drugs may also lead to differences in efficacy. Gefitinib has a slow clearance rate in brain tumor tissue 24 h after administration, and its concentration in brain tumor tissue is significantly higher than that of erlotinib and icotinib. After a single administration in tumor-bearing mice, McKillop et al. found that the 8-h tumor/plasma drug concentration ratio was between 3-fold and 7–8-fold of the concentration at 2 h, which is also an important factor in the antitumor effect of gefitinib [33]. Icotinib has a shorter half-life than either gefitinib or erlotinib, and the drug is rapidly eliminated from tissues. Zhang et al. found that 12 h after administration, organ tissue concentrations of icotinib were reduced to a quarter of the concentration at 1 h post-administration [34]. Furthermore, drug concentrations in brain tumor tissue are much lower than in extracranial tissues. Therefore, along with its rapid elimination from tissues, intermittent administration of icotinib may not allow the drug to reach a concentration high enough to exert its inhibitory effect on tumors.

In this study, the 1-h CSF penetration rate of gefitinib was 1.32±0.37%, which is close to that reported by Chen et al. [18]. The 1-h CSF penetration rate of erlotinib was 0.97±0.31%, which is close to that reported by Lassman et al. [35], and much lower than that reported by Masuda et al. [36]. However, in Masuda et al.'s study, the CSF penetration rate of erlotinib in cancer patients ranged from 2.5–13.3%, indicating that there is a considerable difference between species and between individuals within the same species. The 2-h CSF penetration rate of icotinib was 0.35±0.14%, which was lower than that reported by Fan et al. [12]. In Fan et al.'s study, the 2-h CSF penetration rate of icotinib was 1.4±1.1%; CSF penetration rates among different patients varied greatly, ranging from 0.42% to 4.26%. Fan et al. [12] also combined icotinib with WBRT, which can increase the permeability of the BBB [37]; they therefore speculated that the CSF penetration rate may be lower in icotinib monotherapy. CSF concentration is often used to reflect the ability of EGFR TKIs to penetrate the BBB in a clinical setting and to explain the possible differences in efficacy [38]. Chen et al. [18] found that the effective concentrations of gefitinib in CSF and in brain tumor tissue differed in a brain metastasis mouse model. This phenomenon has been observed not only in EGFR TKIs, and as numerous animal studies have shown, there is a significant difference in drug concentrations in brain extracellular fluid and in CSF; one possible mechanism may be pumping of the drug by BCRP1 and P-gp from the brain parenchyma to CSF through the ventricles of the brain [39, 40]. Because of tumor heterogeneity, there is also a difference in drug concentrations in different areas within the tumor lesion. Sharma et al. found a 3-fold difference in the highest and lowest concentrations of gefitinib in different areas of lesions in a brain glioma mouse model [30]. In the present study, CSF drug concentrations and penetration rates of the three TKIs were significantly lower than those in brain tumor tissues. This indicates that drug concentrations in tumor tissue are the most important factor in determining efficacy. Measuring the concentrations of EGFR TKIs in CSF therefore has limited utility in determining drug concentrations within tumor cells.

This research has certain limitations: Firstly, for the animal model adopted in this study, a method of direct intracranial transplantation was applied rather than an internal carotid injection method for tumor formation [41], which damaged the BBB to a certain extent. However, strict random distribution principles were used in the three study drugs and the baseline situation was consistent, so there was minimal effect on the comparative analysis. The method of injecting PC-9 cells into the internal carotid artery for tumor formation has a tendency to form disseminated miliary brain metastases, i.e. multiple small focal-type metastases [18]. Different from research exploring the mechanism of tumor transplantation and blood metastases as the object of study, this study primarily compared the efficacy of EGFR-TKIs in the treatment of intracranial tumors, for which the direct intracranial transplant method created a more consistent model. Furthermore, whether in drug efficacy studies of metastatic brain tumors or intracranial primary tumors, this type of animal model is widely applied [20, 42, 43]. Secondly, in this study, we did not investigate the potential effect of WBRT on the penetration of EGFR-TKIs into the CSF. WBRT not only directly kills tumor cells, but may also damage endothelial cells, resulting in increased permeability of the BBB and increased penetration of TKIs [44]. Data from retrospective studies have suggested that a combination of EGFR-TKIs and radiotherapy was better than EGFR-TKIs or radiotherapy alone [45, 46]. However, in Fang et al.'s [47] study, no significant change was noted in the CSF/plasma ratios of gefitinib before and after WBRT (2.79±1.47 vs. 2.35±1.74%, p=0.123). Once more, the once-daily icotinib administration method does not mimic the clinical method of administration, so this may not be a true reflection of the drug concentration and penetration rate of icotinib. However, this could not be the explanation for the poor efficacy of icotinib. In a nude mouse model, icotinib showed similar antitumor activity to gefitinib at doses of 60 and 120 mg∙kg−1 [48].

In conclusion, gefitinib and erlotinib exhibited good antitumor activity in a PC-9-luc human heterotopic brain metastasis model, while icotinib activity was similar to that of the control. There are limitations to using the CSF penetration rate to reflect drug efficacy. Distribution differences in brain tumor tissue are important factors that lead to differences in efficacy. A strict clinical controlled study should be designed to further compare efficacy among the three drugs.

## MATERIALS AND METHODS

### Laboratory animals and cells

Eight–ten-week old female SPF BALB/c nude mice weighing 20–24 g were obtained from Beijing Anikeeper Biotechnology, Ltd. (Beijing, China). The PC-9 cell line from a human lung adenocarcinoma cell strain (EGFR 19 del) was purchased from the European Collection of Authenticated Cell Cultures (ECACC, Salisbury, UK). All animal experiments were performed in accordance with the guidelines approved by Institutional Animal Care and Use Committees (IACUCs).

### Reagents and instruments

D-luciferin was obtained from Pharmaron New Drug Technology Co. Ltd. (Beijing, China). Gefitinib hydrochloride (molecular weight 483.36, 99.68% pure) was obtained from Medchem Express (Monmouth Junction, NJ, USA). Erlotinib hydrochloride (molecular weight 429.90, 99.71% pure) and OSI-420 (molecular weight 415.87, 99.05% pure) were obtained from Selleck (Houston, TX, USA). Icotinib hydrochloride (molecular weight 391.42, 99.40% pure) was obtained from Zhejiang Betta Pharmaceutical Industry Ltd. (Hangzhou, China). Instruments included an Agilent 1200 high-performance liquid chromatograph (Agilent Technologies, Santa Clara, CA, USA), and an MS/MS system (SCIEX API4000 liquid chromatography mass spectrometer, Framingham, MA, USA).

### Cell culture

PC-9-luc tumor cells were cultured in RPMI 1640 culture medium with 2 mM L-glutamine (HyClone, GE Healthcare, Little Chalfont, UK) containing 10% inactivated fetal bovine serum (Sigma-Aldrich, St. Louis, MO, USA), 100 U/mL penicillin, and 100 U/mL streptomycin at 37°C in a 5% CO_2_ incubator. Tumor cells in the logarithmic growth phase were inoculated into tumors *in vivo*.

### Tumor cell inoculation and grouping

In accordance with methods reported in the literature [21], a brain metastasis animal model was established through intracranial injection of a solution containing PC-9-luc tumor cells (2 × 10^5^). Thirteen days after inoculation, animals were randomized into four groups: 1) Negative control group; 2) gefitinib (40 mg∙kg^−1^); 3) erlotinib (25 mg∙kg^−1^); 4) icotinib (60 mg∙kg^−1^). TKIs were suspended at the desired concentration in a vehicle containing 0.1% Tween 80 and administered to animals by oral gavage once a day for 4 weeks. The control group received 0.2 mL 0.1% Tween 80. Each group included nine animals. Equivalent conversions of clinical doses to administration doses were based on normalization of body surface area according to “Guidance for Industry Estimating the Maximum Safe Starting Dose in Initial Clinical Trials for Therapeutics in Adult Healthy Volunteers”, FDA, 2005 (https://www.fda.gov/downloads/Drugs/GuidanceComplianceRegulatoryInformation/Guidances/UCM078932.pdf). Administration began on the second day after group assignments.

### Bioluminescent imaging

The IVIS Lumina II small animal *in vivo* imaging system (Caliper Life Sciences, Waltham, MA, USA) was used to perform imaging of mice twice every 10 days. Imaging was performed once before sacrificing. Signal intensity of tumor cell inoculation sites was measured in mice bio-optically and taken as the main indicator for evaluating tumor growth and drug efficacy.

### Specimen collection and tissue processing

On day 40, animals were anesthetized 1 h (n=3), 2 h (n=3), and 24 h (n=3) after drug administration, and blood samples (centrifuged to collect plasma after treatment with anticoagulant) and CSF samples were stored in a freezer at −80°C for pharmacokinetic analysis. Animals underwent systemic perfusion using saline at 4°C, and normal brain and brain tumor samples were then collected for pharmacokinetic analysis.

### Drug concentration measurements

Drug concentrations in plasma, CSF, normal brain, and brain tumor tissue samples were measured using LC-MS/MS (combined drug concentration). Concentrations of gefitinib, erlotinib and its metabolite OSI-420, and icotinib were measured. The ratio of C_CSF_ or C_brain tumor_ to C_plasma_ indicates the rate of drug penetration and distribution: Penetration ratio=(C_CSF_ or brain tumor)/C_plasma_ [18].

### Immunohistochemistry

Parts of brain tumor samples were fixed in formalin and then embedded in paraffin for analysis of pEGFR and Ki-67. After tumor sections were fixed and dehydrated, they were serially cut into sections (thickness: 4 μm). Sections were then dewaxed on a baking sheet and rehydrated for hematoxylin and eosin (H&E) staining. Consecutive sections in which H&E staining confirmed the presence of tumor tissue were dewaxed on a baking sheet, hydrated, and then incubated overnight in EDTA-Tris antigen or citrate buffer, pEGFR (Tyr-1068) (CST-2234, Shanghai Univbio Co., Shanghai, China) antibodies (1:4,000), and Ki-67 (D2H10) (9027S, CST) antibodies (1:200). Secondary antibodies were added and incubated at 22±3°C. The ABC mixture was incubated and colored with DAB, then stained with hematoxylin, dehydrated, and mounted. Image-Pro Plus 6.0 software (Media Cybernetics, Rockville, MD, USA) was used to analyze the immunohistochemistry images. Analyses were performed on all images to obtain the positive IOD values for each image.

### Statistical analysis

SPSS 13.0 software (IBM SPSS, Armonk, NY, USA) was used for statistical analysis. Bioluminescent data are expressed as means ± standard error of the mean (SEM). Additional measurement data are expressed as means ± standard deviation (SD). One-way ANOVA was used for comparisons between groups, and the least significant difference (LSD) test was conducted for homogeneity of variance. Dunnett's T3 test was conducted for heterogeneity of variance. *p*<0.05 indicates a statistically significant difference.

## Abbreviations

BBB: blood-brain barrier
BTB: blood-tumor barrier
EGFR: epidermal growth factor receptor
TKI: tyrosine kinase inhibitor
CNS: central nervous system
WBRT: whole-brain radiotherapy
P-gp: P-glycoprotein
BCRP: breast cancer resistance protein
